# Supplementary material for: Anatomical Targeting of Anticancer Drugs to Solid Tumors Using Specific Administration Routes: Review
Source: Pharmaceutics. 2023 Jun 6;15(6):1664. doi: 10.3390/pharmaceutics15061664 (PMC10301590; doi:10.3390/pharmaceutics15061664)
Supplement: Supplementary file 1 [file pharmaceutics-15-01664-s001.zip › pharmaceutics-2421861-supplementary.pdf]

Supplemental Table S1. Mechanisms of action of the drugs cited in this review

**Drug name**	**Type of drug**	**Mechanism of action**
Cisplatin [1]	Platinum-based drugs	Generates DNA damage by interacting with purine bases in DNA, with subsequent activation of several signaling pathways, ultimately leading to apoptosis.
Doxorubicin [2]	Anthracycline antineoplastic agents	Cytostatic effects attributed to DNA intercalation, DNA binding, and cross-linking, inhibition of topoisomerase II, and induction of apoptosis.
Epirubicin[2]	Anthracycline antineoplastic agents	Cytostatic effects attributed to DNA intercalation, DNA binding, and cross-linking, inhibition of topoisomerase II, and induction of apoptosis.
Miriplatin[3]	Liposoluble platinum complexes	Dichloro[(1R, 2R)-1, 2-cyclohexanediamine-N, N']platinum, the most abundant platinum compound released from miriplatin, induces apoptosis through formation of platinum-DNA adducts.
5-FU[4]	Antimetabolite	Inhibits thymidylate synthase, blocking thymidine formation required for DNA synthesis.
Mitomycin [5]	Anticancer antibiotic	Inhibits DNA replication by inhibiting DNA division and by scission of DNA strands caused by reactive oxygen species.
Oxaliplatin [6]	Platinum-based drug	Generates DNA damage by interacting with purine bases in DNA with subsequent activation of several signaling pathways, ultimately leading to apoptosis.
Carboplatin [7]	Platinum-based drug	Cytotoxic DNA-damaging compound that causes DNA strand breaks, leading to apoptosis.
Methotrexate [8]	Antifolate	Inhibits dihydrofolate reductase, depleting folic acid necessary for nucleic acid synthesis, etc., thereby stopping tumor cell division or killing them.
Docetaxel [9]	Taxane anticancer agents	Promotes and stabilizes assembly of microtubules, preventing their depolymerization, and inhibitsingcancer cell proliferation.
Paclitaxel [9]	Taxane anticancer agents	Promotes and stabilizes assembly of microtubules, preventing their depolymerization, and inhibitsingcancer cell proliferation.
Nedaplatin [10]	Platinum-based drug	Generates DNA damage by interacting with purine bases in DNA, with subsequent activation of several signaling pathways, ultimately leading to apoptosis.
6-mercaptopurine [11]	Purine antagonist	Inhibits de novo purine synthesis and acts as an antiproliferative agent by interfering with protein, DNA and RNA synthesis and promoting apoptosis.
Vincristine [12]	Vinca alkaloid	Induces cytotoxicity by binding to tubulin, resulting in microtubule depolymerization and metaphase arrest.

Cyclophosphamide [13]	Alkylating agent	Preferentially alkylates the N7 position of guanine in DNA, inhibits DNA replication and protein synthesis, and suppresses cancer cell proliferation.
Chimeric antigen receptor T (CAR-T) cells [14]	CAR T cells	CARs are MHC receptor-independent and bind to cell surface-expressed target antigens, leading to T cell activation and potent anti-tumor effects.
Cytarabine [15]	Antimetabolite	Cytotoxic due to incorporation of ara-CTP into DNA and inhibition of DNA synthesis.
Topotecan [16]	Topoisomerase inhibitor	Inhibits DNA replication by inhibiting topoisomerase I, killing cancer cells.
Irinotecan [16]	Topoisomerase inhibitor	Inhibits DNA replication by inhibiting topoisomerase I, killing cancer cells.
Transferrin receptor ligand-targeted toxin conjugate (Tf-CRM107) [17]	Conjugated Toxins	A conjugate of transferrin and a point mutant of diphtheria toxin that selectively kills cells expressing high levels of the transferrin receptor.
TP-38 [17]	Conjugated Toxins	A chimeric protein that contains a fragment of *Pseudomonas* exotoxin and TGF-α. It binds to and selectively kills cells expressing high levels of epidermal growth factor receptor (EGFR).
IL13-PE38QQR [18]	Conjugated Toxins	A recombinant chimeric cytotoxin that contains IL-13 and a fragment of *Pseudomonas* exotoxin. It binds to and selectively kills cells expressing high levels of interleukin-13R alpha2 receptors.
Reovirus [17]	Oncolytic viruses	A genetically modified reovirus that preferentially infects and kills cancer cells over non-malignant cells.
LIPO-HSV-1-tk [17]	Liposomes	A liposomal vector that contains the herpesvirus thymidine kinase (tk) gene. Upon delivery of this gene, cells become sensitized to ganciclovir, a nucleoside analog that is clinically used as an antiviral.
CpG-28 [17]	Oligodeoxynucleotides	Acts as a bacterial pathogen-associated molecular pattern (PAMP) analog that interacts with TLR9 to stimulate immune responses activating anti-tumor immunity.
AP-12009 [17]	Oligodeoxynucleotides	Binds to and suppresses cells expressing the malignant factor, TGFβ-2.
^{131}I-chTNT-1/B MAb (Cotara) [19]	Chimeric antibody	A genetically engineered chimeric monoclonal antibody that binds to the DNA-histone H1 complex, and carries ^{131}I, which delivers beta particles locally, as well as gamma radiation to kill adjacent tumor cells.
Bevacizumab [20]	Molecular targeting drugs	Blocks angiogenesis and suppresses tumor cell proliferation by inhibiting tumor vascular endothelial growth factor A (VEGF-A).
Gemcitabine [21]	Antimetabolite	Taken up by cancer cells and phosphorylated by dioxycytidine kinase (DCK) to form gemcitabine triphosphate, which is incorporated into cellular DNA, inhibiting nuclear replication, and suppress cancer cell division and proliferation.

Catumaxomab [22]	Molecular targeting drugs	A trifunctional monoclonal antibody with two antigen-binding sites, EpCAM and CD3, and a functional Fc domain that activates a complex antitumor immune reaction through various effector functions, such as anti-body-dependent cellular cytotoxicity, phagocytosis, and T cell-mediated cytotoxicity
SD-101 [23]	TLR9 agonists	Activates immune cells around the tumor (myeloid cells, B cells, etc.) to release various cytokines (tumor necrosis factor (TNF), IL-12, interferons (IFN) α/β/γ and IL-6, etc.) and promoting anti-tumor immunity.
Tilsotolimod [23]	TLR9 agonists	Activates immune cells around the tumor (myeloid cells, B cells, etc.) to release various cytokines (tumor necrosis factor (TNF), IL-12, interferons (IFN) α/β/γ and IL-6, etc.) and promoting anti-tumor immunity.
PV-10 [23]	Oncolytic viruses	Genetically modified polio virus that preferentially infects and kills cancer cells over non-malignant cells.
Talimogene laherparepvec (T-VEC) [23]	Oncolytic viruses	Genetically modified herpes simplex virus-1 (HSV-1) that preferentially infects and kills cancer cells over non-malignant cells.
CAVATAK [24]	Oncolytic viruses	Genetically modified coxsackievirus A21. It preferentially infects and kills cancer cells over non-malignant cells.
HF10 [24]	Oncolytic viruses	Genetically modified herpes simplex virus-1 (HSV-1). preferentially infects and kills cancer cells over non-malignant cells.
Pexastimogene devacirepvec (Pexa-Vec) [23]	Oncolytic viruses	Genetically modified vaccinia virus. It preferentially infects and kills cancer cells over non-malignant cells.
PVS-RIPO [24]	Oncolytic viruses	Genetically modified polio/rhinovirus. It preferentially infects and kills cancer cells over non-malignant cells.
DNX-2401 [23]	Oncolytic viruses	Genetically modified adenoviruses. It preferentially infects and kills cancer cells over non-malignant cells.

1. Ghosh, S. Cisplatin: The first metal based anticancer drug. *Bioorg Chem* **2019**, *88*, 102925, doi:10.1016/j.bioorg.2019.102925.
2. Gyöngyösi, M.; Lukovic, D.; Zlabinger, K.; Spannbauer, A.; Gugerell, A.; Pavo, N.; Traxler, D.; Pils, D.; Maurer, G.; Jakab, A.; et al. Liposomal doxorubicin attenuates cardiotoxicity via induction of interferon-related DNA damage resistance. *Cardiovasc Res* **2020**, *116*, 970-982, doi:10.1093/cvr/cvz192.
3. Hanada, M.; Baba, A.; Tsutsumishita, Y.; Noguchi, T.; Yamaoka, T.; Chiba, N.; Nishikaku, F. Intra-hepatic arterial administration with miriplatin suspended in an oily lymphographic agent inhibits the growth of tumors implanted in rat livers by inducing platinum-DNA adducts to form and massive apoptosis. *Cancer Chemother Pharmacol* **2009**, *64*, 473-483, doi:10.1007/s00280-008-0895-3.
4. Wigmore, P.M.; Mustafa, S.; El-Beltagy, M.; Lyons, L.; Umka, J.; Bennett, G. Effects of 5-FU. *Adv Exp Med Biol* **2010**, *678*, 157-164, doi:10.1007/978-1-4419-6306-2_20.
5. Tomasz, M.; Palom, Y. The mitomycin bioreductive antitumor agents: cross-linking and alkylation of DNA as the molecular basis of their activity. *Pharmacol Ther* **1997**, *76*, 73-87, doi:10.1016/s0163-7258(97)00088-0.
6. Riddell, I.A. Cisplatin and Oxaliplatin: Our Current Understanding of Their Actions. *Met Ions Life Sci* **2018**, *18*, doi:10.1515/9783110470734-007.

7. Kelland, L. The resurgence of platinum-based cancer chemotherapy. *Nat Rev Cancer* **2007**, *7*, 573-584, doi:10.1038/nrc2167.
8. Laqué-Rupérez, E.; Ruiz-Gómez, M.J.; de la Peña, L.; Gil, L.; Martínez-Morillo, M. Methotrexate cytotoxicity on MCF-7 breast cancer cells is not altered by exposure to 25 Hz, 1.5 mT magnetic field and iron (III) chloride hexahydrate. *Bioelectrochemistry* **2003**, *60*, 81-86, doi:10.1016/s1567-5394(03)00054-9.
9. Ojima, I.; Lichtenthal, B.; Lee, S.; Wang, C.; Wang, X. Taxane anticancer agents: a patent perspective. *Expert Opin Ther Pat* **2016**, *26*, 1-20, doi:10.1517/13543776.2016.1111872.
10. Ota, K. [Nedaplatin]. *Gan To Kagaku Ryoho* **1996**, *23*, 379-387.
11. Fernández-Ramos, A.A.; Marchetti-Laurent, C.; Poindessous, V.; Antonio, S.; Laurent-Puig, P.; Bortoli, S.; Loriot, M.A.; Pallet, N. 6-mercaptopurine promotes energetic failure in proliferating T cells. *Oncotarget* **2017**, *8*, 43048-43060, doi:10.18632/oncotarget.17889.
12. Thomas, D.A.; Sarris, A.H.; Cortes, J.; Faderl, S.; O'Brien, S.; Giles, F.J.; Garcia-Manero, G.; Rodriguez, M.A.; Cabanillas, F.; Kantarjian, H. Phase II study of sphingosomal vincristine in patients with recurrent or refractory adult acute lymphocytic leukemia. *Cancer* **2006**, *106*, 120-127, doi:10.1002/cncr.21595.
13. Bordin, D.L.; Lima, M.; Lenz, G.; Saffi, J.; Meira, L.B.; Mésange, P.; Soares, D.G.; Larsen, A.K.; Escargueil, A.E.; Henriques, J.A.P. DNA alkylation damage and autophagy induction. *Mutat Res* **2013**, *753*, 91-99, doi:10.1016/j.mrrev.2013.07.001.
14. Sterner, R.C.; Sterner, R.M. CAR-T cell therapy: current limitations and potential strategies. *Blood Cancer J* **2021**, *11*, 69, doi:10.1038/s41408-021-00459-7.
15. Wang, L.M.; White, J.C.; Capizzi, R.L. The effect of ara-C-induced inhibition of DNA synthesis on its cellular pharmacology. *Cancer Chemother Pharmacol* **1990**, *25*, 418-424, doi:10.1007/bf00686052.
16. You, F.; Gao, C. Topoisomerase Inhibitors and Targeted Delivery in Cancer Therapy. *Curr Top Med Chem* **2019**, *19*, 713-729, doi:10.2174/1568026619666190401112948.
17. Nwagwu, C.D.; Immidisetti, A.V.; Jiang, M.Y.; Adeagbo, O.; Adamson, D.C.; Carbonell, A.M. Convection Enhanced Delivery in the Setting of High-Grade Gliomas. *Pharmaceutics* **2021**, *13*, doi:10.3390/pharmaceutics13040561.
18. Vogelbaum, M.A.; Sampson, J.H.; Kunwar, S.; Chang, S.M.; Shaffrey, M.; Asher, A.L.; Lang, F.F.; Croteau, D.; Parker, K.; Grahn, A.Y.; et al. Convection-enhanced delivery of cintredekin besudotox (interleukin-13-PE38QQR) followed by radiation therapy with and without temozolomide in newly diagnosed malignant gliomas: phase 1 study of final safety results. *Neurosurgery* **2007**, *61*, 1031-1037; discussion 1037-1038, doi:10.1227/01.neu.0000303199.77370.9e.
19. Hdeib, A.; Sloan, A. Targeted radioimmunotherapy: the role of ^{131}I-chTNT-1/B mAb (Cotara) for treatment of high-grade gliomas. *Future Oncol* **2012**, *8*, 659-669, doi:10.2217/fon.12.58.
20. Garcia, J.; Hurwitz, H.I.; Sandler, A.B.; Miles, D.; Coleman, R.L.; Deurloo, R.; Chinot, O.L. Bevacizumab (Avastin®) in cancer treatment: A review of 15 years of clinical experience and future outlook. *Cancer Treat Rev* **2020**, *86*, 102017, doi:10.1016/j.ctrv.2020.102017.
21. Pandit, B.; Royzen, M. Recent Development of Prodrugs of Gemcitabine. *Genes (Basel)* **2022**, *13*, doi:10.3390/genes13030466.
22. Bokemeyer, C. Catumaxomab--trifunctional anti-EpCAM antibody used to treat malignant ascites. *Expert Opin Biol Ther* **2010**, *10*, 1259-1269, doi:10.1517/14712598.2010.504706.
23. Melero, I.; Castanon, E.; Alvarez, M.; Champiat, S.; Marabelle, A. Intratumoural administration and tumour tissue targeting of cancer immunotherapies. *Nat Rev Clin Oncol* **2021**, *18*, 558-576, doi:10.1038/s41571-021-00507-y.
24. Zawit, M.; Swami, U.; Awada, H.; Arnouk, J.; Milhem, M.; Zakharia, Y. Current status of intralesional agents in treatment of malignant melanoma. *Ann Transl Med* **2021**, *9*, 1038, doi:10.21037/atm-21-491.
